# Quantitative mass spectrometric analysis to unravel glycoproteomic signature of follicular fluid in women with polycystic ovary syndrome

**DOI:** 10.1371/journal.pone.0214742

**Published:** 2019-04-04

**Authors:** Krutika Patil, Soujanya Yelamanchi, Manish Kumar, Indira Hinduja, T. S. Keshava Prasad, Harsha Gowda, Srabani Mukherjee

**Affiliations:** 1 Department of Molecular Endocrinology, National Institute for Research in Reproductive Health, Indian Council of Medical Research, Mumbai, India; 2 Institute of Bioinformatics, International Technology Park, Bangalore, India; 3 P.D. Hinduja National Hospital and Medical Research Centre, Mumbai, India; 4 Center for Systems Biology and Molecular Medicine, Yenepoya Research Centre, Yenepoya (Deemed to be University), Mangalore, India; Peking University Third Hospital, CHINA

## Abstract

Polycystic ovary syndrome (PCOS) is a complex endocrinopathy affecting women of reproductive age, and whose etiology is not well understood yet. In these women, the follicular growth is arrested at preantral stage leading to cyst formation, consequently resulting in anovulatory infertility in these women. As the follicular fluid provides the conducive microenvironment for the growth of oocytes, molecular profiling of the fluid may provide unique information about pathophysiology associated with follicular development in PCOS. Post-translational addition of oligosaccharide residues is one of the many modifications of secreted proteins influencing their functions. These glycoproteins play a significant role in disease pathology. Despite glycoproteins having such essential functions, very limited information is available on their profiling in human reproductive system, and glycoproteomic profile of follicular fluid of women with PCOS is yet unexplored. In the present study, we performed a comparative glycoproteomic analysis of follicular fluid between women with PCOS and controls undergoing in vitro fertilization, by enrichment of glycoproteins using three different lectins viz. concanavalin A, wheat germ agglutinin and Jacalin. Peptides generated by trypsin digestion were labeled with isobaric tags for relative and absolute quantification reagents and analyzed by liquid chromatography tandem mass spectrometry. We identified 10 differentially expressed glycoproteins, in the follicular fluid of women with PCOS compared to controls. Two important differentially expressed proteins- SERPINA1 and ITIH4, were consistently upregulated and downregulated respectively, upon validation by immunoblotting in follicular fluid and real-time polymerase chain reaction in granulosa cells. These proteins play a role in angiogenesis and extracellular matrix stabilization, vital for follicle maturation. In conclusion, a comparative glycoproteomic profiling of follicular fluid from women with PCOS and controls revealed an altered expression of proteins which may contribute to the defects in follicle development in PCOS pathophysiology.

## Introduction

Polycystic ovary syndrome (PCOS) is the most common endocrine disorder affecting 6% to 15% of reproductive age women, however, its etiology and pathogenesis remains uncertain till now [1]. Classically it is characterized by altered gonadotropin secretion, irregular menses, enlarged ovaries with multiple cysts on ultrasound imaging, together with hyperandrogenism (hirsutism, acne, alopecia), insulin resistance, obesity and abnormal glucose metabolism. Women with PCOS tend to have a higher risk of developing type 2 diabetes mellitus, dyslipidemia, coronary artery disease and endometrial cancer [2,3] in later life. Follicular growth is arrested at preantral stage and the eventual formation of fluid filled cysts leads to anovulatory infertility in these women. Several studies have indicated that the oocyte fertilization capacity is compromised in women with PCOS [2–4].

Follicular fluid is present in the antrum of growing follicle and consists of secretions from granulosa cells (GCs) and exudates from plasma through thecal capillaries. It contains crucial growth factors, proteins, steroids, polysaccharides etc. and plays an essential role in follicular growth, oocyte maturation and ovulation. Secretions from GCs also promote oocyte maturation and competence via paracrine signaling and transfer of nutrient across gap junctions [5]. Thus, the composition of follicular fluid proteins may indirectly reflect the oocyte quality and predict subsequent embryo development competence [6,7]. Alterations in the proteomic signature of follicular fluid could reveal the molecular mechanisms involved in healthy follicle development and oocyte maturation and also help to decipher the underlying pathophysiology of ovarian disorders [8].

Majority of the secreted proteins are glycosylated [9]. Glycosylation occurs by an enzymatic addition of carbohydrate moieties onto the amide group of asparagine (N-linked glycosylation) or on the hydroxyl group of serine or threonine residues (O-linked glycosylation). In humans, addition of oligosaccharide chains on to proteins alter protein folding, its trafficking, stability, hydrolytic properties and subsequently its function [10–12]. Glycoproteins modulate cellular events such as cell–cell communication, cell–matrix interactions, cell migration and signaling; all these processes are also vital for the development of a mature oocyte and its subsequent ovulation. Global analysis of glycoproteins is important to understand disease biology and is considered immensely valuable for noninvasive detection of clinical biomarkers which may subsequently be developed into diagnostic and therapeutic targets [13]. Alteration in either the glycosylation sites or heterogeneity of glycan residues have been reported to lead to the genesis and progression of disease such as neurodegenerative disorder, CVD, inflammatory diseases and cancer [14,15].

A comparative proteomic study between follicular fluid of women with PCOS and control women by our group has shown a dysregulation of 186 proteins, many of which are known to undergo glycomodification [8], highlighting the importance of studying glycoproteins in pathophysiology of PCOS. However, the glycoprotein composition of follicular fluid of women with PCOS has not yet been studied. Hence, we carried out comparative glycoproteome analysis of follicular fluid from women with PCOS and controls.

Glycoproteins are less abundant and therefore require efficient enrichment and detection techniques. Current developments of mass spectrometry (MS) technology for proteomic analysis and emerging glycoproteins enrichment methods such as multi-lectin affinity technique have facilitated analysis of differential glycoproteins in normal and diseased states. Each type of lectin has an affinity to bind to a specific glycan structure, and thus a combination of three lectins concanavalin A (Con A), wheat germ agglutinin (WGA) and jacalin (JAC) were used in the present study to enrich glycoproteins from follicular fluid. In the present study, we have undertaken a quantitative approach based on isobaric tags for relative and absolute quantitation (iTRAQ) followed by liquid chromatography mass spectrometry (LC-MS/MS) analysis to compare glycoproteomic profiles of follicular fluid from women with PCOS with that of healthy controls in an effort to identify any alteration of glycoproteins associated with PCOS.

## Materials and methods

Enriched glycoproteins from follicular fluid of PCOS and control samples were used for iTRAQ labeling in duplicates. Subsequently, the samples were digested with trypsin and subjected to strong cation exchange (SCX) and strong anion exchange (SAX) fractionation, followed by LC-MS/MS analysis. The samples were analyzed on Orbitrap Fusion tribid mass spectrometer (Thermo Fisher Scientific Inc., Bremen, Germany).

### Study subjects and sample collection

This study has been approved by the Institutional Ethics Committee of ICMR-National Institute for Research in Reproductive Health (ICMR-NIRRH) and Institutional Review Board of P. D. Hinduja National Hospital. The study was carried out at NIRRH as per the ethical guidelines with written informed consent obtained from all participants. This study comprised a total of 57 participants of which 27 were diagnosed with PCOS, according to Rotterdam consensus criteria [16] and 30 women were age and BMI-matched, regularly menstruating healthy women undergoing IVF at P. D. Hinduja National Hospital. The diagnosis of PCOS included the presence of at least two of the following three features: i) oligomenorrhoea and/or anovulation, ii) clinical and/or biochemical signs of hyperandrogenism, and iii) polycystic ovaries on ultrasound [16]. Women with other related disorders like non-classical congenital adrenal hyperplasia, thyroid dysfunction, androgen secreting tumors, and hyperprolactinemia were not included. The healthy women underwent IVF either because their spouses were diagnosed with male factor infertility or as oocyte donors with regular menses, normal ovaries on ultrasound and no signs of hyperandrogenism. All participants underwent controlled ovarian hyperstimulation using GnRH agonist and recombinant FSH (long protocol). Briefly, GnRH agonist, (0.5 mg of buserlin acetate) was administered daily starting from the 21^st^ day of the previous menstrual cycle. Once down-regulation was confirmed via ultrasound and serum estradiol measurements, ovarian stimulation was carried out with administration of recombinant follicle-stimulating hormone (r-hFSH), with reducing GnRH analog dose to half. On maturation of the follicles (diameter, 16–18 mm), recombinant human chorionic gonadotropin (hCG, 10,000 IU) was administered, and oocyte retrieval was performed 34–36 h later by transvaginal ultrasound-guided aspiration. During oocyte retrieval, ~5 ml macroscopically clear follicular fluid, lacking visible blood contamination was collected from all participants. Further, these follicular fluids were processed as described previously [17], and stored at -80°C until use. After oocyte retrieval, the GCs were collected and separated on a Ficoll gradient (HiMedia, India), washed with phosphate buffer saline (PBS), and cells were lysed with qiazol to obtain total RNA (Qiagen, Germany). Following RNA extraction, gene expression studies were carried out.

### Biochemical and hormonal assays

Follicular fluid samples were collected on the day of ovum pick up (d-OPU) from women with PCOS and control groups. Samples were assayed for estradiol (E_2_), progesterone (P_4_), testosterone (TT) and sex hormone binding globulin (SHBG) using commercially available ELISA kits (Diagnostics Biochem Canada Inc., Dorchester, Ontario, Canada). The basal levels of follicular stimulating hormone (FSH), luteinizing hormone (LH), thyroid-stimulating hormone (TSH), anti-Mullerian hormone (AMH), prolactin, and E_2_, measured before and after the administration of hCG during IVF and ovarian characteristic, were obtained from clinical records.

### Glycoprotein enrichment

Total protein concentration of follicular fluid from 15 controls and 15 PCOS samples were measured using the Bicinchoninic (BCA) method of protein estimation (Pierce, Rockford, USA). Approximately 670 μg of protein from each sample was pooled to obtain a total of 10 mg protein for controls and PCOS separately.

Glycoprotein enrichment of follicular fluid was carried out by using a mixture of three different agarose-bound lectins, Con A, WGA and JAC (Vector laboratories, USA). Con A preferentially binds alpha-mannosyl groups, whereas WGA has a higher specificity for N-acetylglucosamine-containing glycoproteins and JAC interacts readily with mannose, glucose, and N-acetylneuraminic acid. Since these lectins show affinity for a broad spectrum of glycoproteins, we used a mixture of the above three lectins and the bound glycoproteins were eluted using corresponding mixture of sugars.

Equivalent volumes of approximately 200μl of each type of agarose bound lectins were re-suspended together and further split into two equal halves and used for the enrichment of glycoproteins from PCOS and control follicular fluid samples, respectively. The lectins were washed three to four times with ice cold 1X PBS by centrifuging at 1,000 rpm for 2 min at 4°C. Subsequently, follicular fluids from each group were loaded onto the beads separately and the total volume was raised to 5 ml with 50 mM Tris-HCl and 150 mM NaCl, (pH 7.5). The samples were incubated overnight at 4°C on a rotor for binding of the glycoproteins. Following overnight incubation, beads were centrifuged at 1000 rpm at 4°C for 5 min and the supernatant were removed. Subsequently, the beads were washed three times with ice cold 1X PBS and the bound glycoproteins were eluted using a mixture of sugars. Briefly, 200 mM α-methyl mannoside for Con A, 500 mM N-acetyl-D-glucosamine for WGA and 100 mM melibiose for JAC in Tris buffer, pH 7.5 were used for the elution of glycoproteins. The beads were incubated with sugar solutions for 30 min on a rocker at room temperature; the supernatants were collected by centrifuging at 1500 rpm for 10 min at 4°C and the whole procedure was repeated twice. The pooled supernatants of control and PCOS samples were concentrated by using 3 kDa MWCO filters (Amicon, Millipore, Ireland). The final protein amount was estimated by BCA protein assay kit (Pierce, Rockford, USA) and stored at −20°C until further use.

### iTRAQ labeling and peptide fractionation

Equal amounts of protein from both PCOS and control sample groups, were subjected to denaturation with 2% SDS, reduction with 5 mM DTT and subsequent alkylation with 20 mM Iodoacetamide. Further, digestion was performed using modified sequencing grade Trypsin (Promega, Madison, WI, USA) at 37°C overnight in a 1:20 enzyme to substrate ratio. Peptides were then labeled with iTRAQ reagents according to the manufacturers’ instructions (iTRAQ Reagents Multiplex kit; Applied Biosystems/ MDS Sciex, Foster City, CA). Samples were labeled in technical replicates as follows: control samples with 114 and 115 reporter tags and PCOS samples with 116 and 117 reporter tags respectively. All labeled samples were then pooled and vacuum-dried.

Dried peptides were subjected to stage tip-based SAX and SCX fractionation as previously described [18]. Briefly, SAX and SCX material were punched separately into 200 μl tips and the stage tips were centrifuged at a speed of 1000g to 2000g during fractionation. The peptides were reconstituted in SAX pH 12 buffer (20 mM acetic acid, 20 mM phosphoric acid, and 20 mM boric acid) and elution was performed separately with SAX buffers of pH 2, pH 5, pH 9 and pH 12, where the pH was adjusted with 1M NaOH. In case of SCX stage tip fractionation, the peptides were reconstituted in 1% trifluoroacetic acid (TFA) and the 6 fractions were eluted with different concentrations of SCX buffer (50mM, 75mM, 125mM, 200mM, 300 mM ammonium acetate, 20% ACN and 0.5% formic acid) and buffer X containing 5% ammonium hydroxide, 80% ACN. All the SCX and SAX fractions were cleaned using C18 stage tips prior to mass spectrometry analysis.

#### LC-MS/MS analysis

Peptides were reconstituted in 0.1% formic acid and analyzed on an Orbitrap Fusion tribrid mass spectrometer (Thermo Fisher Scientific Inc., Bremen, Germany) interfaced with Easy-nLC 1000 system. Peptides were first enriched on a trap column (75 μm × 2 cm, C18 material 5 μm, 100 Å) using solvent A (5% ACN, 0.1% formic acid) at a flow rate of 3 μL/min and resolved on an in-house packed analytical column (75 μm × 20 cm, C18 material 3 μm, 120 Å) using a linear gradient of 7–35% solvent B (95%ACN, 0.1% formic acid) at a flow rate of 300 nL/min. The nanospray source maintained at 2 kV ion spray voltage. MS data was acquired in a data dependent manner with full scans acquired using the Orbitrap mass analyzer at a mass resolution of 120,000 at 200 m/z and MS/MS scans acquired at a mass resolution of 30,000 at 200 m/z. In addition to four SAX and six SCX fractions, we also generated four other fractions by analyzing the peptides at four different mass ranges at MS level i.e. 300–800 m/z, 300–1000 m/z, 800–2000 m/z, 1000–2000 m/z. Data dependent MS/MS scans were triggered using Top speed mode with a cycle time of 3 sec and a minimum intensity threshold of 10,000. The fragmentation was carried out using higher-energy collision dissociation (HCD) with 32% NCE and step collision energy of 5%. The fragmented precursors were dynamically excluded for 30 sec to prevent repeated MS/MS analysis of precursor ions. The automatic gain control for FT-MS was set to 2x10^5^ ions and for FT-MS/MS 5x10^4^ ions with a maximum ion injection time of 100 ms for MS and 200 ms for MS/MS analysis. Lock mass option (m/z 445.12002) was enabled for accurate mass measurements.

#### Data analysis

MS/MS raw data was analyzed using SEQUEST algorithm in Proteome Discoverer version 2.1 software (Thermo Fisher Scientific Inc., Bremen, Germany). Human Uniprot protein database with known contaminants was included for the database searching. Search parameters included trypsin as the enzyme with maximum 2 missed cleavage allowed. Oxidation of methionine was set as a dynamic modification while carbamidomethylation at cysteine and iTRAQ modification at N-terminus of the peptide and at lysine were set as static modifications. Precursor and fragment mass tolerance were set to 10 ppm and 0.05Da, respectively. False discovery rate of 1% at peptide level was used to filter the confident peptide identifications. Reporter ion quantitation node was used for relative expression pattern of proteins based on the relative intensities of reporter ions for the corresponding peptide. Proteins with ratios 1.4-fold increase or decrease between controls and PCOS were considered to be up or down regulated, respectively. The mass spectrometry proteomics data have been deposited to the ProteomeXchange Consortium via the PRIDE [19] partner repository with the dataset identifier PXD012731.

### Immunoblotting

Validation of differentially expressed proteins was carried out by immunoblotting. 15 μg of follicular fluid protein from 7 women with PCOS and 7 controls were resolved by 10% SDS-PAGE and trans-blotted onto a PVDF membrane (Pall corporation, Pensacola, Fl, USA). The membranes were blocked with 5% non-fat dry milk followed by incubation for 16 hours with mouse anti-human SERPINA1 antibody (1:10000, ab9400 Abcam, MA, USA) or goat anti-human ITIH4 (1:500, ab92338 Abcam, MA USA), respectively. After incubation, blots were washed with PBS, incubated with secondary antibody, horseradish peroxidase (HRP)-labelled anti-mouse (1:10000) or anti-goat (1:10000) antibody, (Dako, CA, USA), and incubated for 1 hour. Detection was carried out using ECL prime detection reagents (Amersham ECL Prime western blotting detection kit, GE Healthcare, UK). The band intensities were quantified using Image Lab Software 6.0.1 (Bio-rad, USA) against Coomassie Brilliant Blue (CBB) stained total loading control.

### Real time-PCR

RNA extraction from GCs was performed using Qiagen miRNA easy kit (Qiagen, Hilden, Germany) according to manufacturers’ instructions. RNA estimation was carried out using a nanodrop (Gen5 Software). cDNA was synthesized using high capacity cDNA reverse transcription kit with RNase inhibitor (Applied Biosystems, CA, USA). qRT-PCR analysis were done for SERPINA1 and ITIH4 gene using Taqman gene expression assay (Thermo Fisher Scientific, USA) following the manufactures’ instructions, in GCs obtained from 10 controls and 10 women with PCOS. The mRNA abundance was normalized to expression of housekeeping gene, 18S rRNA and represented as fold change values by Δ threshold cycle (Ct) 2^-(ΔΔCt)^ calculation. Values were expressed as mean ± S.D.

### Statistical analysis

Data was analysed using Mann-Whitney U test to compare PCOS and controls (GraphPad prism 5.0 software). Differences in datasets were considered significant when the p-value was <0.05.

## Results

### Characteristics of the study participants

The clinical, hormonal, and biochemical characteristics of women with PCOS and controls, undergoing controlled ovarian stimulation (COH) are outlined in Tables 1 and 2. Baseline serum LH levels, LH/FSH ratio and AMH levels were significantly higher with lower FSH in the PCOS group when compared to controls (Table 1). On the day of hCG administration, women with PCOS showed significantly higher serum E_2_ levels (Table 1). On the d-OPU, follicular fluid levels of E2, TT, T, bioavailable testosterone (bio-T) and free androgen index (FAI) were significantly high and SHBG, P_4_, levels were significantly low in women with PCOS compared to controls (Table 2). Furthermore, analysis of oocyte characteristics showed significantly more preovulatory follicle number in PCOS and no difference in, mature and percent MII oocytes between both groups. However, percentages of fertilized MII oocytes were found to be significantly lower in women with PCOS compared to controls.

**Table 1:** Baseline characteristics and parameters assessed before and after initiation of COH in study participants undergoing IVF.

	Control (n = 30), Median (IQR)	PCOS (n = 27), Median (IQR)	P value
**Age, years**	28 (21–35)	28 (24–36)	0.122
**BMI, kg/m**^**2**^	22.85 (18.30–39.13)	25.1 (19.40–34.80)	0.061
**Basal LH levels, mIU/mL**	3.6 (2.630–13.30)	8.730 (4.290–19.5)	0.005
**Basal FSH levels, mIU/mL**	5.725 (4.3–9.350)	5.295 (2.340–9.50)	0.045
**Basal LH/FSH ratio**	0.6551 (0.4496–3.093)	1.639 (0.612–3.61)	0.008
**Prolactin, (ng/mL)**	11.65 (4.1–17.63)	11.00 (7.86–24.82)	0.656
**TSH, μIU/mL**	1.36 (0.2–3.2)	1.65 (0.52–4.3)	0.417
**AMH, ng/mL**	3.005 (0.57–7.86)	8.5 (2–21.50)	0.002
**Hormones and oocyte parameters recorded after initiation of COH**			
**E**_**2**_ **(ng/mL) before hCG administration**	1.962 (0.815–2.968)	2.158 (0.836–6.782)	0.707
**E**_**2**_ **(ng/mL) on day of hCG administration**	3.185 (0.931–5.533)	4.535 (2.376–9.065)	0.034
**Preovulatory follicles (n)**	17 (5–29)	20 (10–40)	0.034
**Mature or MII oocyte (n)**	13 (5–25)	16 (9–38)	0.064
**% MII oocyte**	91.59 (42.86–100)	88.12 (55.56–100)	0.263
**Fertilized MII oocyte (n)**	12 (2–24)	11 (3–32)	0.454
**% Fertilized MII oocyte (% ROF)**	89.90 (42.86–100)	81.48 (20–94.44)	0.035

Data are represented as median-interquartile range (IQR). Statistical comparison was done using Mann Whitney U test. P values <0.05 were considered statistically significant. Abbreviations: BMI, body mass index; LH, luteinizing hormone; FSH, follicle stimulating hormone; TSH, thyroid stimulating hormone; AMH, Anti-mullerian hormone; ROF, rate of fertilization.

**Table 2:** Hormonal parameters measured in follicular fluid of study participants on the day of oocyte pick up (d-OPU).

Follicular fluid	Control (n = 30),	PCOS (n = 27),	P value
	Median (IQR)	Median (IQR)	
**E**_**2**_ **(ng/mL)**	943.3 (715–1250)	1065 (800–1756)	0.009
**P**_**4**_ **(μg/mL)**	10.91 (4.365–15.07)	7.810 (2–13)	0.01
**TT (ng/dL)**	203.7 (160.5–350.3)	441 (270–698.2)	<0.0001
**SHBG (nmol/L)**	196.9 (140–290)	159 (100–201)	<0.0001
**Free T (pmol/L)**	35.74 (26.72–43.03)	94.21 (47.89–121.5)	<0.0001
**Bio-T (nmol/L)**	0.8415 (0.6315–1.006)	2.210 (1.128–3.286)	<0.0001
**FAI**	3.881 (2.894–4.616)	9.976 (5.205–14.77)	<0.0001

Data are represented as median-interquartile range (IQR). Statistical comparison was done using Mann Whitney U test. P values <0.05 were considered significant Abbreviations: E_2_, estradiol; P_4_, progesterone; TT, total testosterone; SHBG, sex hormone binding globulin; Free T, free testosterone; Bio-T, bioavailable testosterone; FAI (free androgen index).

### Quantitative analysis of glycoproteins in follicular fluid

**Fig 1:**
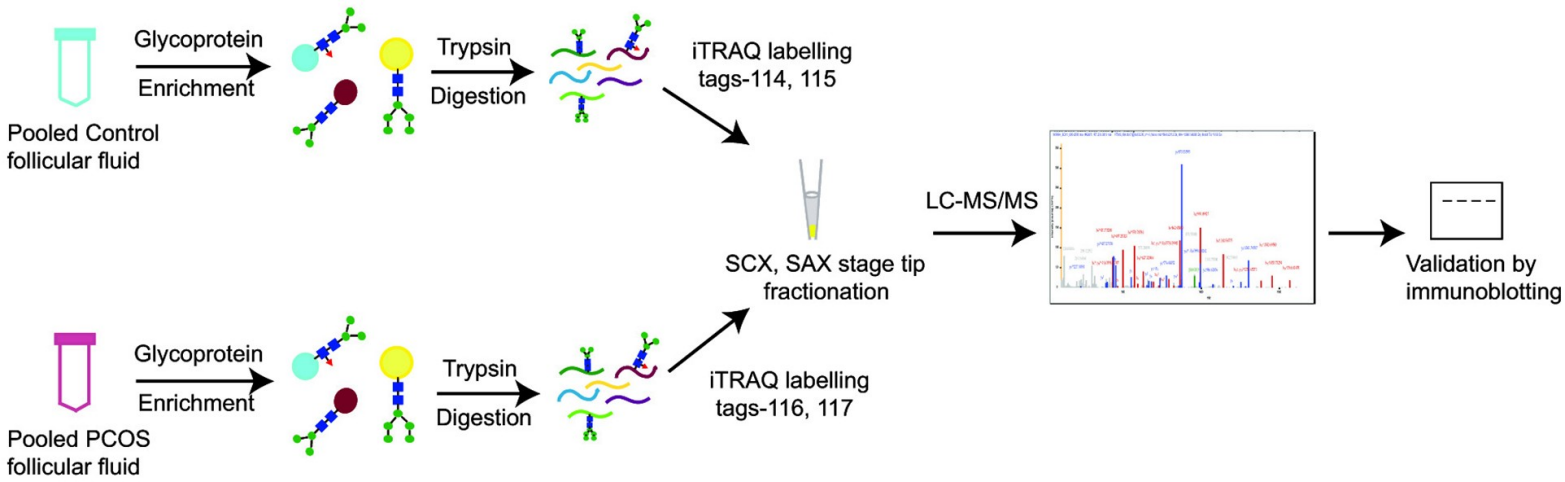
Schematic representation of the workflow of glycoprotein enrichment. By iTRAQ-based strategy employed for the comparison of follicular fluid proteome from healthy controls and women with PCOS.

**Fig 2:**
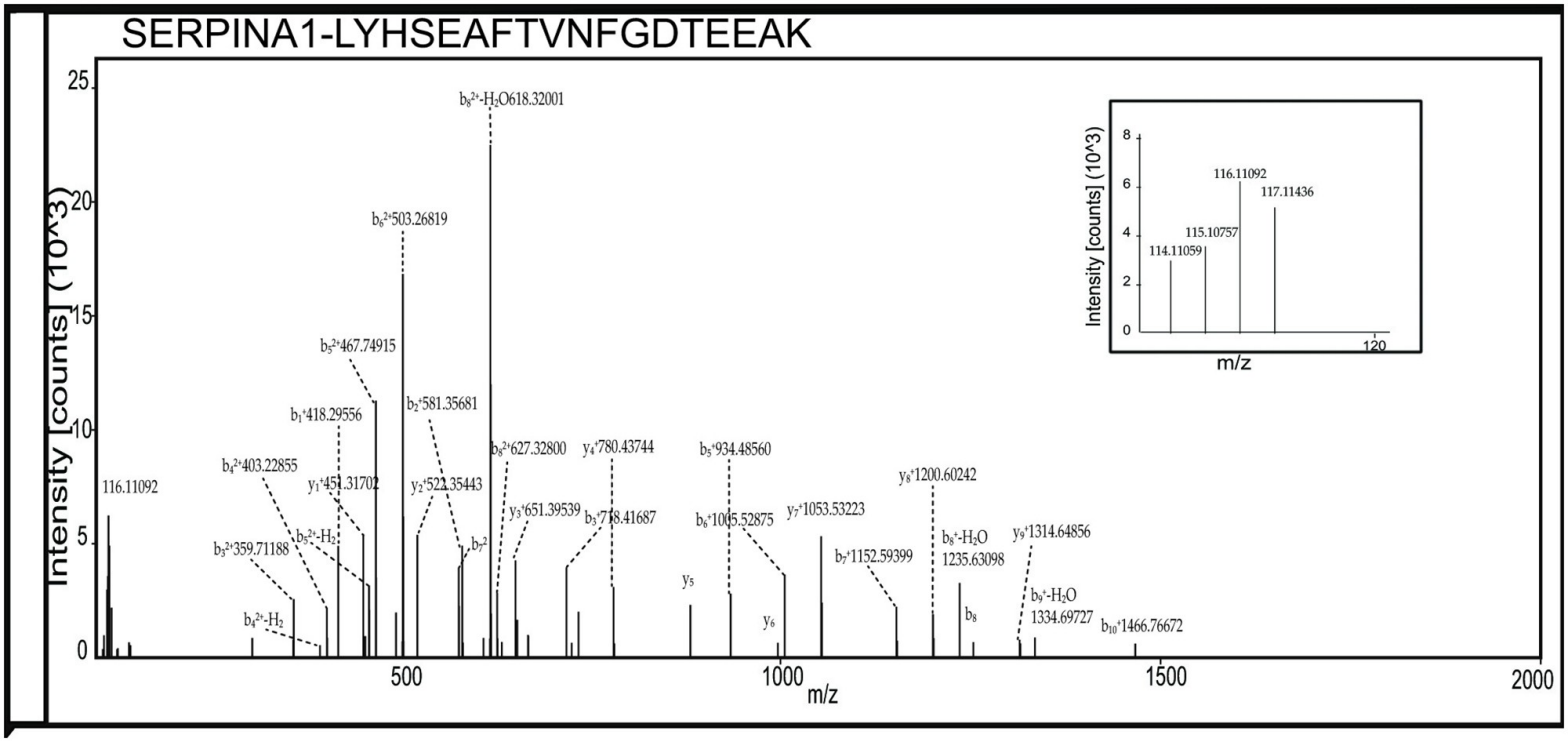
Representative MS/MS spectra of peptides from upregulated protein SERPINA1. Inset shows relative intensities of reporter ions.

**Fig 3:**
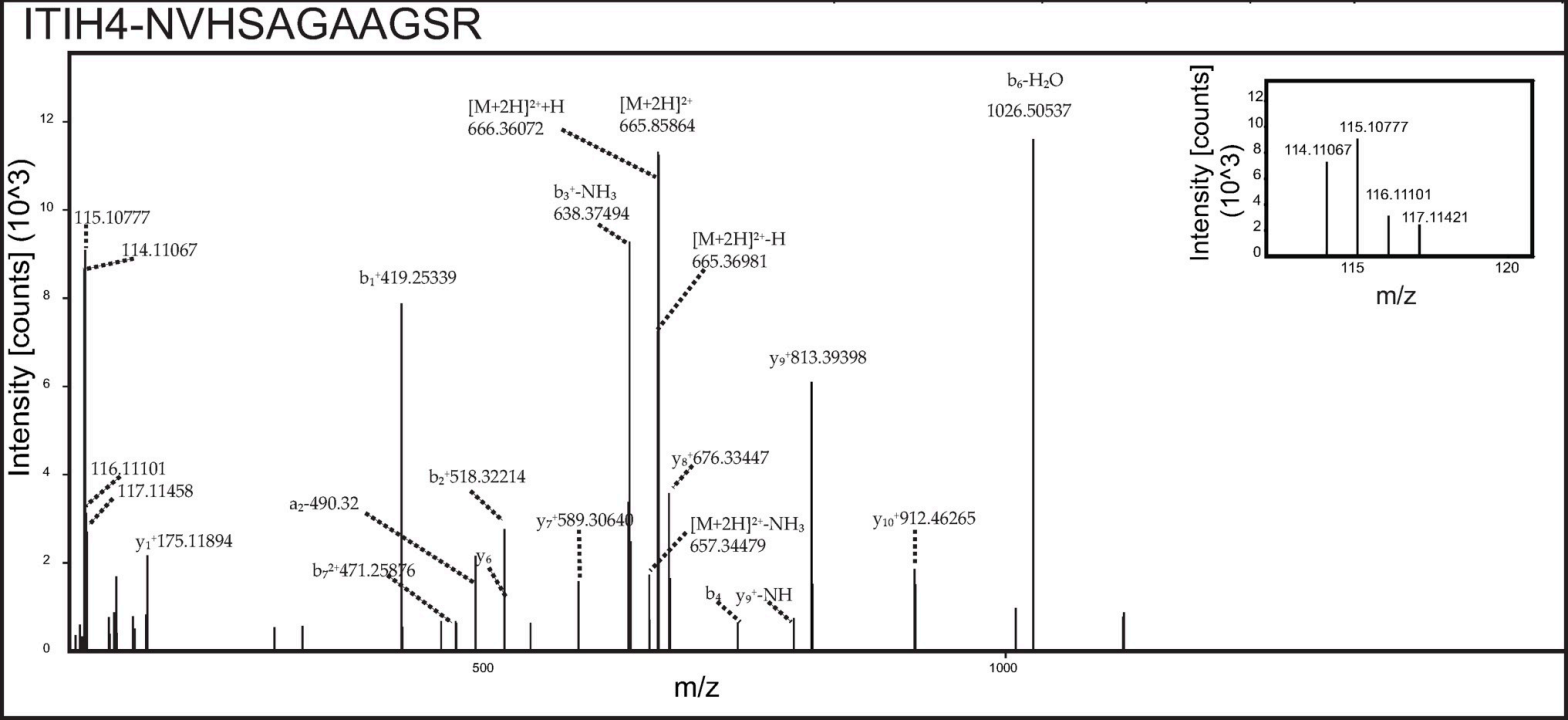
Representative MS/MS spectra of peptides from downregulated protein ITIH4. Inset shows relative intensities of reporter ions.

To investigate the glycoproteins in the follicular fluid of women with PCOS compared to controls, glycoprotein enriched follicular fluid samples were labeled with iTRAQ reagents and analyzed by LC-MS/MS (Fig 1). In our study, a total of 1,880 peptide groups belonging to 282 proteins were identified (S1 Table). The identified peptide and protein data were fetched using high peptide confidence (1% FDR) and rank one peptide match filters. Through bioinformatics analysis, 74% of proteins were annotated as N-linked and/or O-linked glycoproteins in the Uniprot/ Genecard database. Proteins that showed significant changes, ±1.4 fold in magnitude between PCOS and control women were considered as differentially expressed. Ten differentially regulated glycoproteins were identified, and listed in Table 3. Three proteins were found to be overexpressed in follicular fluid of women with PCOS, while 7 proteins were down regulated, when compared to the controls. Up-regulated proteins were alpha-1-antitrypsin precursor (SERPINA1), hemoglobin subunit beta and alpha, while down-regulated proteins included chondroadherin like protein (CHADL), dopamine beta-hydroxylase (DBH), inter-alpha-trypsin inhibitor heavy chain H4 (ITIH4), inhibin alpha chain (INHA), Ig gamma-4 chain C region, tumor necrosis factor receptor superfamily member 1A (TNFRSF1A) and vitamin K-dependent protein C (PROC). Representative labeled MS/MS spectra of peptides from proteins SERPINA1 and ITIH4 are shown in Figs 2 and 3 respectively.

**Table 3:** List of differential proteins obtained from the follicular fluid glycoproteins enriched using a lectin-based approach and subsequent LC-nano-ESI-MS/MS.

Description	Gene ID	Abundance Ratio: PCOS/Control
Chondroadherin-like protein	*CHADL*	0.367
Dopamine beta-hydroxylase	*DBH*	0.562
Inter-alpha-trypsin inhibitor heavy chain H4	*ITIH4*	0.596
Ig gamma-4 chain C region	*IGHG4*	0.672
Inhibin alpha chain	*INHA*	0.694
Tumor necrosis factor receptor superfamily member 1A	*TNFRSF1A*	0.706
Vitamin K-dependent protein C	*PROC*	0.709
Alpha-1-antitrypsin	*SERPINA1*	1.431
Hemoglobin subunit beta	*HBB*	1.465
Hemoglobin subunit alpha	*HBA2; HBA1*	1.694

### Validation of differentially expressed proteins

**Fig 4:**
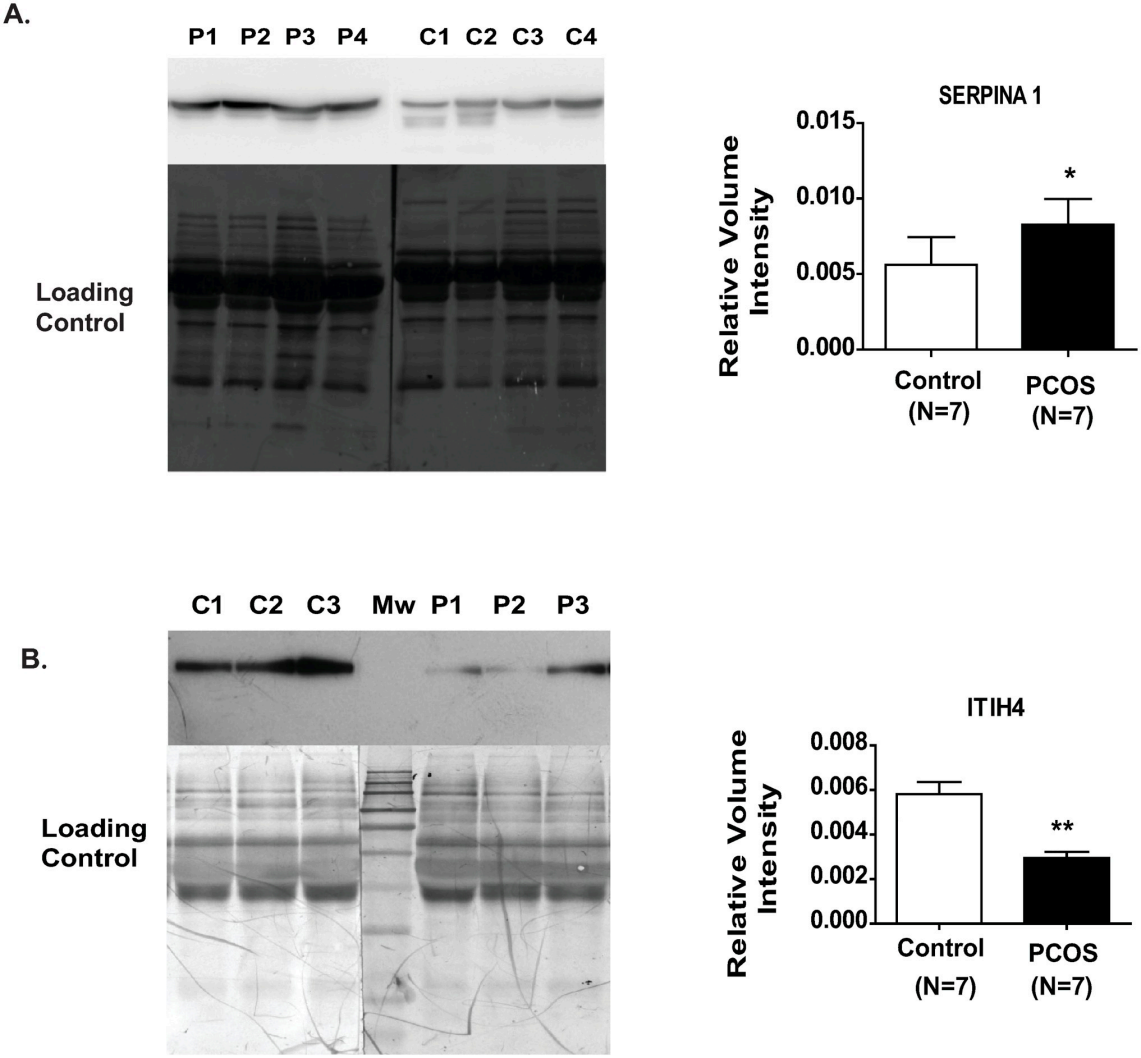
Validation of SERPINA1 upregulation (A) and ITIH4 downregulation (B), at protein level in follicular fluid by immunoblotting. Each lane was loaded with 10 μg follicular fluid protein obtained from women with PCOS and controls. Lanes designated as C are control samples and as P are PCOS follicular fluid. The total protein load was used as loading control. Data are represented as mean ± S.D. Statistical comparison was done using Mann Whitney U tests. P values <0.05 are considered statistically significant.

Two important glycoproteins were found to be differentially expressed in follicular fluid upon validation by immunoblotting. Upregulated glycoprotein SERPINA1 was confirmed to increase while downregulated glycoprotein, ITIH4 showed a definitive decrease, in the follicular fluid of women with PCOS, when compared to that of control women (Fig 4A and 4B).

**Fig 5:**
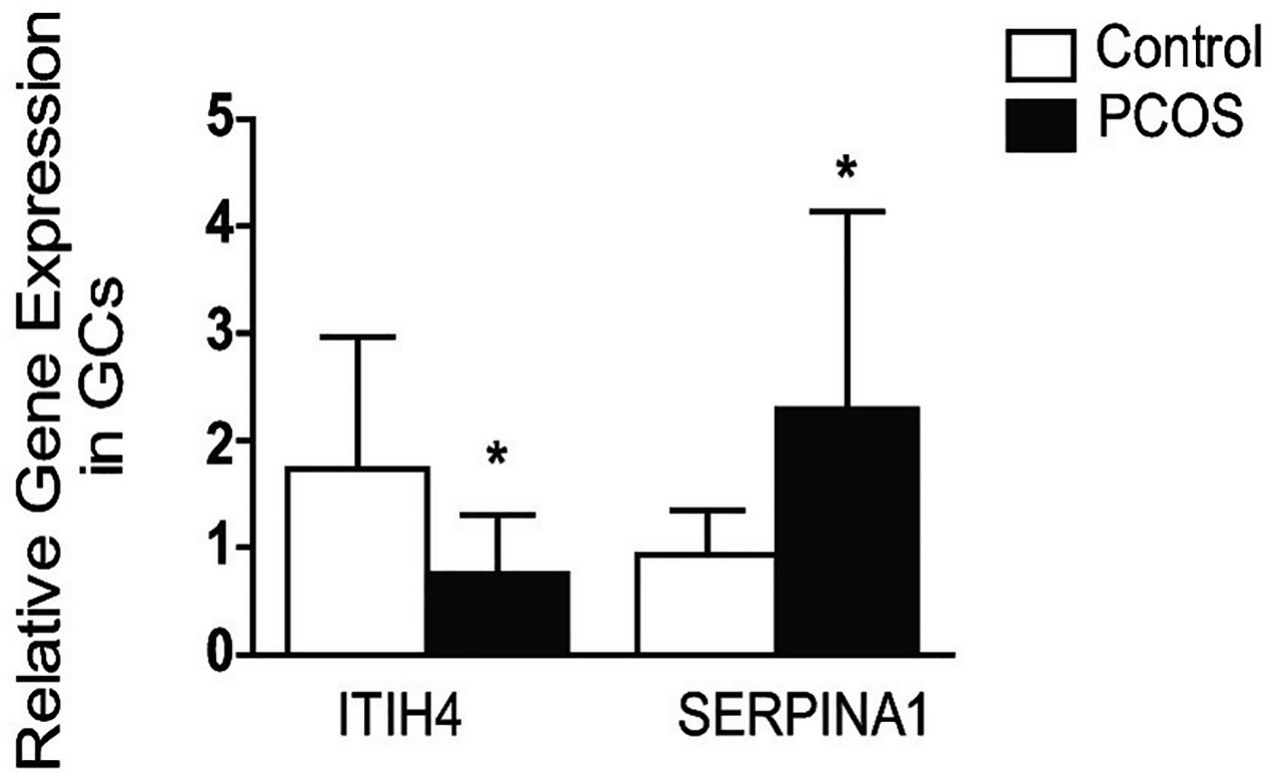
Relative gene expressions of ITIH4 and SERPINA1 in the GCs of women with PCOS and control. Data are represented as mean ± S.D. Statistical comparison was done using Mann Whitney U tests. P values <0.05 are considered statistically significant.

The proteins present in the follicular fluid consist of secreted proteins from GCs and those percolated from serum. Since the differential levels of these proteins in follicular fluid may be a direct result of their altered expressions in GCs, we investigated the transcript expression of both of these proteins in GCs by qPCR. In, congruence with the protein levels in follicular fluid, the gene expression of ITIH4 is downregulated in GCs while SERPINA1 is upregulated in GCs of women with PCOS (Fig 5).

## Discussion

The composition of follicular fluid provides information about the microenvironment of the growing oocyte essential for follicle development. Glycoproteins are vital constituents of the cell membrane and extra cellular matrix (ECM), involved in biological processes including intracellular transport, protein folding and stability, cell-cell and receptor-ligand interaction and immune response. Changes in glycosylation may alter a biological function, folding and stability of proteins, which in turn may contribute to disease progression [20]. We hereby, present the first report on the use a quantitative proteomic technique to perform a comparative glycoprotein expression profiling of follicular fluid from women with PCOS versus healthy controls. Our study has led to the identification of 282 glycoproteins present in the follicular fluid of women with PCOS compared to controls, of which, 10 proteins were found to be differentially regulated. Further, we confirmed up and downregulation of SERPINA1 and ITIH4, respectively in follicular fluid by immunoblotting.

SERPINA1, one of the serine protease inhibitors in serum, is an acute phase protein, with a documented role in the inhibition of neutrophil elastase in lungs, modulating inflammation and apoptosis [21]. Apart from its tissue specific functions, SERPINA1 is an inhibitor of various tissue remodeling and angiogenesis-promoting proteases including, plasmin, thrombin, trypsin and plasminogen activator. Plasminogen activator, catalyzes the conversion of plasminogen to plasmin by proteolytic cleavage. Plasmin in turn activates vascular endothelial growth factor, basic fibroblast growth factor and matrix metalloproteases, all of which play an important role in angiogenesis. SERPINA1 exerts its anti-angiogenic effect by reducing the pro-angiogenic activity of plasmin, which in turn affects the subsequent activation of the above-mentioned pro-angiogenic factors. Additionally, it also interferes with ECM degradation, another important activity essential for angiogenesis. In our earlier comparative proteomic study, we found downregulation of plasminogen and an upregulation of SERPINA1 in the follicular fluid of women with PCOS [8]. Plasminogen activator inhibitor, the main regulator of plasminogen activator has been reported to be increased in the serum of women with PCOS [22,23]. All this may lead to lower plasmin level and activity in women with PCOS.

Since SERPINA1 is a serum derived protein; its high levels in follicular fluid may be due to its higher abundance in serum, which may percolates into the follicular fluid. Alternatively, high follicular fluid levels of SERPINA1 may also be on account of its increased expression and subsequent secretion from GCs. Therefore, we measured the transcript levels of SERPINA1 in GCs which was significantly upregulated in women with PCOS, suggesting that GCs also contribute towards the increased level of SERPINA1 in follicular fluid of this women. Our results are also in corroboration with findings of Kaur et al, who reported higher SERPINA1 transcripts in GCs of PCOS compared to controls by microarray analysis [24]. The transcript and protein of SERPINA1 have been reported to be over expressed in the endometrial tissue of women with PCOS [25]. Altogether our results indicate that increased SERPINA1 may be involved in altered angiogenesis during follicle development, thereby contributing towards PCOS pathology. However, due to the non-availability of serum samples from study participants on the d-OPU, we could not measure the serum concentration of SERPINA1.

Another differentially regulated protein, is ITIH4, a 120-kDa glycoprotein, readily cleaved by plasma kallikrein into various smaller fragments. ITIH4 belongs to the inter-alpha-trypsin inhibitors family of plasma serine protease inhibitors, and has the von Willebrand factor A domain, which may help to mediate its interaction with other proteins [26]. Although its exact function is not known, it is reported to act as an acute phase protein and helps in the stabilization of ECM by interacting with hyaluronic acid (HA), a major component of ECM [26,27]. Following LH surge during the preovulatory period, the organization and expansion of cumulus oocyte complex (COC) occurs, which is essential for the developmental changes required for oocyte maturation. During this expansion, the cumulus cells secretes large amounts of HA in the ECM. HA consists of negatively charged, linear glycosaminoglycans, whose proteolytic products have pro-angiogenic functions. Tumor necrosis factor alpha-inducible protein 6 (TNFAIP6) carries out transesterification reaction, i.e. the covalent transfer of heavy chains of ITIH4 to HA, thus, it acts as a catalyst for the formation of stable complexes of ITIH4 and HA [27]. Hess et al, showed that, inter alpha trypsin inhibitor polypeptides play an important role in the expansion of COC in mouse oocyte [28]. Alpha-1-microglobulin/ bikunin precursor (AMBP) codes for light chain present in inter-alpha-trypsin inhibitors chains. Previously, we showed a down regulation of TNFAIP6 and AMBP [8] in PCOS follicular fluid, while in the present study, we found down-regulation of ITIH4 in both follicular fluid and GCs. Altogether these observations indicate that downregulation of these proteins may lead to ECM destabilization, which may in turn adversely affect the COC expansion, and may contributes towards compromised oocyte quality and fertilization competence, as seen in women with PCOS.

Blood vessel formation and regression are critical components of folliculogenesis. Regulated angiogenesis is required for follicle development, oocyte maturation, ovulation and corpus luteum formation. Available reports suggest that angiogenesis is altered in PCOS [29,30]. We have observed dysregulation of both SERPINA1 and ITIH4 which play a role in angiogenesis and ECM stabilization, implying they may be contributing towards altered angiogenesis in the follicles of women with PCOS. In our earlier proteomics study, we found differential expression of many angiogenic proteins [8]. However, further studies are needed to substantiate these result.

Another interesting protein which we found to be downregulated is DBH, involved in conversion of dopamine (DA) into norepinephrine (NE). Both are important brain neurotransmitters, which have also been detected in the ovary. DA has been reported to be upregulated in PCOS follicular fluid [31,32], however the existing reports on NE are controversial [31,33]. Also, DA has been found to be higher in GCs of PCOS, whose metabolism is correlated with the generation of reactive oxygen species in women with PCOS, resulting in higher oxidative stress [32]. The increase in VEGF in ovarian thecal and stromal compartments of women with PCOS may be driven by the dysregulated DA/ DA receptor 2 signaling axis, which negatively regulate VEGF secretion. This may probably results in local increase of VEGF levels, and increased vascularization of the theca and stroma in the ovaries of women with PCOS [34]. Also, patients having a rare homozygous mutation of *DBH* gene, showed impaired cardiovascular autonomic regulation, enhanced glucose-stimulated insulin secretion, and insulin resistance [35], the co-morbidities also observed in women with PCOS. Hence, we propose that downregulation of DBH may alter the DA metabolism, and the eventual derangement in the DA/ DA receptor 2 signaling which may alter vascularization of follicles in women with PCOS.

CHADL, an ECM protein, which was also found to be downregulated in the follicular fluid of women with PCOS in our study. Only a limited information is available regarding the expression and function of this protein. Tillgren et al., observed its expression in developing cartilaginous tissues and cartilage. It was found to be associated with collagen and modulates collagen fibrillogenesis and possibly plays a role in the formation of a stable ECM [36]. A proteomic study on the ECM of cardiovascular tissues of a porcine model of ischemia injury also found this protein [37]. Till now there are no available reports on the presence of this protein in ovarian tissue. As CHADL was not found in the plasma protein database, this protein is most likely synthesized in the ovary and secreted in the follicular fluid [38]. As collagen and ECM proteins play important role in follliculogenesis, down regulation of CHADL along with downregulation of several other collagen and ECM protein which we found in our earlier study may affect follicular growth in women with PCOS.

TNF-α is a pro-inflammatory cytokine, expressed by the oocytes, theca cells, GCs, and corpus luteum, playing an important role in regulating steroidogenesis, folliculogenesis, ovulation, luteinization, fertility and insulin resistance [39]. Actions of TNF-α is mediated via two receptors, TNFRSF1A and TNFRSF1B, among which most biological effects of TNF-α, cytotoxicity, steroidogenesis and proliferation occur via TNFRSF1A activation. Both receptors are present in the GCs [40]. TNFRSF1A knockout mice exhibit early puberty, early senescence and poor fertility despite having a normal estrous cycles [41]. In women with PCOS, a higher level of TNF- α has been reported in the serum and follicular fluid [42–45], however no reports are available on the expression of TNFRSF1A. We have detected a lower expression of TNFRSF1A in PCOS follicular fluid as compared to controls. Higher levels of TNF- α and lower TNF- α receptor in women with PCOS, is indicative of a dysregulation in the TNF-α signaling cascade, eventually might contributing to the pathogenesis of PCOS.

Another protein found to be down-regulated in our study is INHA, which is the α subunit of inhibin A and B protein complex. It is a member of TGFβ superfamily, and is secreted by the granulosa and thecal cells in the ovary. It is a multifunctional glycoprotein whose major function is to provide a negative feedback on FSH secretion at the pituitary gland. Additionally, it is an important modulator of reproductive functions playing a vital role in follicular maturation, cell differentiation, and oocyte development. An earlier study showed that RNAi-mediated knockdown of INHA increased apoptosis in GCs and decreased fertility in mice [46]. Further, INHA level in follicular fluid and its transcript expression in GCs of women with PCOS is reported to be lower as compared to controls [47,48], which may be one of the reasons for its altered paracrine control on folliculogenesis in PCOS. Our results also support earlier findings of downregulation in INHA levels in PCOS.

## Conclusion

In the present study, we investigated the glycoprotein profile of follicular fluid from women with PCOS compared to control wherein we found a differential expression of 10 glycoproteins and validated the expression of the two important glycoproteins, SERPINA1 and ITIH4. Though the number of differentially expressed glycoproteins is small, the identified glycoproteins play critical roles in angiogenesis and ECM stabilisation. Hence, the alteration of these proteins may partially affect follicular development in PCOS. Further studies are necessary to investigate the follicular angiogenesis aspect of PCOS, as altered angiogenesis may lead to follicular arrest, compromised oocyte growth and defect in corpus luteum formation commonly seen in women with PCOS. Also study of glycan structures and their glycosylation sites would reveal more insights of follicular pathophysiology. This study has limited depth which can be overcome, at least in part by, increasing sample size and by the use of more extensive protocols for the glycoprotein enrichment of follicular fluid that would improve protein coverage. The current findings will help to broaden our knowledge of the glycoproteins present in follicular fluid and their differential expression which may be useful for understanding the pathophysiology of PCOS.

## Supporting information

S1 Table List of all proteins obtained from follicular fluid glycoproteins enrichment using Lectin-based approach and LC-nano-ESI-MS/MS. (XLSX)
